# Young’s double-slit experiment with undulator vortex radiation in the photon-counting regime

**DOI:** 10.1038/s41598-023-49825-4

**Published:** 2023-12-27

**Authors:** Shin-Ichi Wada, Hiroyuki Ohta, Atsushi Mano, Yoshifumi Takashima, Masaki Fujimoto, Masahiro Katoh

**Affiliations:** 1 Graduate School of Advanced Science and Engineering, Hiroshima University, Higashi-Hiroshima, 739-8526 Japan; 2 Faculty of Science, Hiroshima University, Higashi-Hiroshima, 739-8526 Japan; 3 Synchrotron Radiation Research Center, Nagoya University, Nagoya, 464-8603 Japan; 4 Institute for Molecular Science, Okazaki, 444-8585 Japan; 5 Hiroshima Synchrotron Radiation Center, Hiroshima University, Higashi-Hiroshima, 739-0046 Japan; 6 Present Address: Technical Office, School of Engineering, Nagoya University, Nagoya, 464‑8603, Japan

**Keywords:** Optical physics, Optical physics

## Abstract

Young’s double-slit interference experiments with undulator vortex radiation were conducted, focusing on photon-counting regime. To isolate the second harmonic radiation in the ultraviolet range emitted from the helical undulator and achieve successful counting measurements, an ultranarrow bandpass filter was utilized under an extremely low-current mode of the electron storage ring. It was observed that the photon spots on the detector, after passing through the double slits, appeared to be randomly distributed. However, upon integrating these photon spots, it was confirmed that interference fringes with characteristic features of optical vortices, such as dark and broken/distorted stripes in the center, were formed. The reproducibility of these interference fringes was confirmed by calculating the optical path difference for the optical vortex reaching the double slits, as well as the optical path difference resulting from normal double-slit interference. Consequently, these findings indicate that even in the state of a single photon, the radiation emitted spontaneously by a high-energy electron in spiral motion possesses the nature of an optical vortex, characterized by a spiral wavefront.

## Introduction

The existence of optical vortices (OVs), which exhibit a spiral wavefront unlike the flat or spherical wavefront of ordinary light, has garnered significant attention over the past few decades. In 1992, Allen et al. conducted a theoretical investigation on Laguerre–Gaussian beams and established that electromagnetic waves with spiral wavefronts can be solutions to Maxwell's equations^1^. One intriguing aspect of OVs is their association with orbital angular momentum (OAM), suggesting a potential avenue for advancing our understanding of the interaction between light and matter. To explore the equivalence of OAM in light, atoms, and molecules, several research endeavors have been pursued. For example, spectroscopic measurements have been conducted to identify new selection rules in electronic transitions induced by OVs^2^. Additionally, studies have explored the trapping of microparticles (optical tweezers)^3,4^, and the transfer of helicity to materials^5,6^ through the physical influence of the helical structure of OVs, along with enantioselective crystal nucleation^7^ and OAM dichroism^8^. The beam profile characteristic of OVs, which features zero intensity at the center, has found application in stimulated emission depletion (STED) microscopy, which was recognized with the 2014 Nobel Prize in Chemistry^9^. The investigation of the distinctive phase structure and OAM carried by light has become a vibrant area of research in particle manipulation, imaging, classical and quantum optical communication, and other related fields^10,11^.

Various techniques have been employed to generate OVs in the visible light wavelength range, involving transformations of Gaussian laser beams in the fundamental TEM_00_ mode. Examples include the utilization of a spiral phase plate^12^, a spatial light modulator^13^, a q-plate optical device^14^, and computer holography^15^. Recent theoretical^16–18^ and experimental^18–20^ studies have established that the harmonic components of electromagnetic waves emitted by electrons in spiral motion passing through a helical undulator naturally possess a helical phase structure. Helical undulator radiation can be understood as the Lorentz transformation of electromagnetic radiation from circularly moving electrons, and it is this motion which leads to the vortex properties of the radiation^17,18^. The fundamental frequency component is circularly polarized but its topological charge is zero. The (*l* + 1)th harmonic frequency component is also circularly polarized but its topological charge is *l*. This may be expressed as that the (*l* + 1)th harmonic carries spin angular momentum of 1 and orbital angular momentum of *l*. Unlike in the generation of OVs by modulating laser beams, OVs from electrons in spiral motion are generated directly at the stage of the emission of light. Depending on physical parameters such as the energy and radius of motion of the electrons, the radiation covers entire wavelengths from radio waves to gamma rays, and is a phenomenon universally present in nature. Consequently, OVs hold the potential for universal existence and are anticipated to broaden the scope of research in this domain.

Katoh et al. demonstrated the helical phase structure of radiation from relativistic electrons in spiral motion using a Young's double-slit diffraction experiment^18^. In the experiment involving the second harmonic component from the helical undulator, distinct diffraction patterns with a broken and distorted structure at the center emerged, deviating from typical interference fringes. This observation confirmed the helical wavefront and the presence of a phase singularity at the center, similar to results observed using a laser-generated beam^21^. In contrast, the fundamental component from the undulator produced linear diffraction fringes in the same experiment. Consequently, questions arise regarding the generation of OVs by individual electrons in helical orbits and whether each photon possesses a spiral wavefront structure. These queries naturally arise after obtaining evidence of OVs from the helical undulator, as the aforementioned measurements characterize OVs within a photon population, whether it be in the form of a radiation beam or pulse.

Young's double-slit interference experiment is renowned for its ability to showcase the wave nature of light. While typically conducted with high light intensity and the occurrence of interference between two light fluxes, experiments using extremely weak light in a single-photon state can demonstrate the wave–particle duality of light. A notable study conducted at the Central Research Laboratory of Hamamatsu Photonics K.K. exemplifies this concept^22^. In this experiment, photons were incident on a double slit with a sufficiently long distance between successive photons, surpassing the coherence length of the photons to prevent interference between them. The formation of interference fringes, resulting from the integration of photon spots detected on a two-dimensional photon-counting imager consisting of a photocathode, a microchannel plate (MCP), and a position-sensitive detector, signifies that a single detected photon passes through the double slits simultaneously and undergoes self-interference. This compelling evidence affirms the dual nature of a single photon as both a particle and a wave. Within the scope of this paper, we have conducted Young's double-slit experiments in the photon-counting regime to verify the aforementioned nature of single vortex radiation from a helical undulator.

## Results and discussion

**Figure 1:**
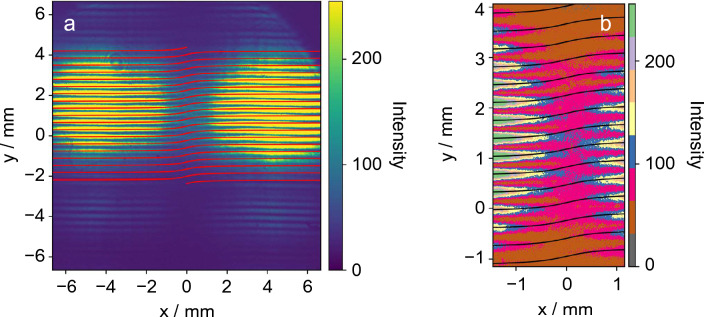
Interference images obtained using Young’s double-slit with undulator vortex radiation. (**a**) Interference fringes measured by CCD camera with 0.5 s exposure time. Red lines indicate calculated fringes using Eq. (1) (*l* = 1). (**b**) The central dark area in (**a**) is enlarged with different color tones to illustrate clear breaks/distortions of fringes that are in good agreement with the black curves obtained using the same equation.

The experiments were conducted at the BL1U undulator beamline of the UVSOR-III electron storage ring^23^, operating in an extremely low-current mode (refer to “Methods” for detailed information). The undulator was adjusted to produce circularly polarized fundamental radiation with a wavelength of 710 nm. The light, along with the second harmonic radiation at 355 nm, was extracted from the vacuum beamline through a quartz window into atmosphere. Only the 355 nm light, which underwent significant attenuation via an ultranarrow bandpass filter, was allowed to pass through a horizontally positioned double slit. The double slit was aligned such that the optical axis intersected the center, and the interference fringes were initially captured using a standard charge-coupled device (CCD) camera. Figure 1a illustrates the results obtained with a 0.5 s exposure time, while Fig. 1b presents an enlarged view of the central region with a distinct color tone. The figure clearly depicts fringes arising from the interference of the double slits, as well as the presence of a singularity characterized by mismatched fringes in the dark central region. This outcome conclusively demonstrates that the interference patterns produced by circularly polarized radiation from the helical undulator exhibit the characteristic features of OVs, as previously reported^18,21^. The darkness and breaks in the fringes arise from the phase singularity (zero intensity) and the phase difference resulting from the spiral structure of the wavefront, respectively. In terms of the orientation of the captured image, with the *x*-axis aligned with the horizontal direction (i.e., the direction of the slits) and the *y*-axis aligned with the vertical direction, the interference fringes generated along the *x*-axis on the CCD screen can be described by the following equation (refer to “Methods” for further details).

1 $$\begin{array}{c}y=\frac{L\lambda }{d}\left[m+\frac{l}{2\pi }\left\{{{\text{tan}}}^{-1}\left(\frac{x}{{y}_{1}}\right)-{{\text{tan}}}^{-1}\left(\frac{x}{{y}_{2}}\right)\right\}\right],\end{array}$$

where *L* is the distance from the double slit to the camera screen, $\lambda$ is the wavelength, *y*_1_ and *y*_2_ are the distances of the two slits from the optical axis (thus the distance between the slits is ${y}_{1}-{y}_{2}$), and *m* and *l* are integers; in particular, *l* is the topological charge of OVs. The provided equation reveals that the Young's interference fringes, generated at a spacing of *Lλ/d*, experience distortion near the optical axis due to the phase difference originating from the spiral wavefront. In Fig. 1, the interference fringes calculated by incorporating the experimental parameters and *l* = 1 for the second harmonic are depicted as thin lines. Remarkably, the obtained measurement results align well with the anticipated characteristics of the interference fringes when a phase singularity exists between the two slits. This agreement further supports the validity of the findings.

**Figure 2:**
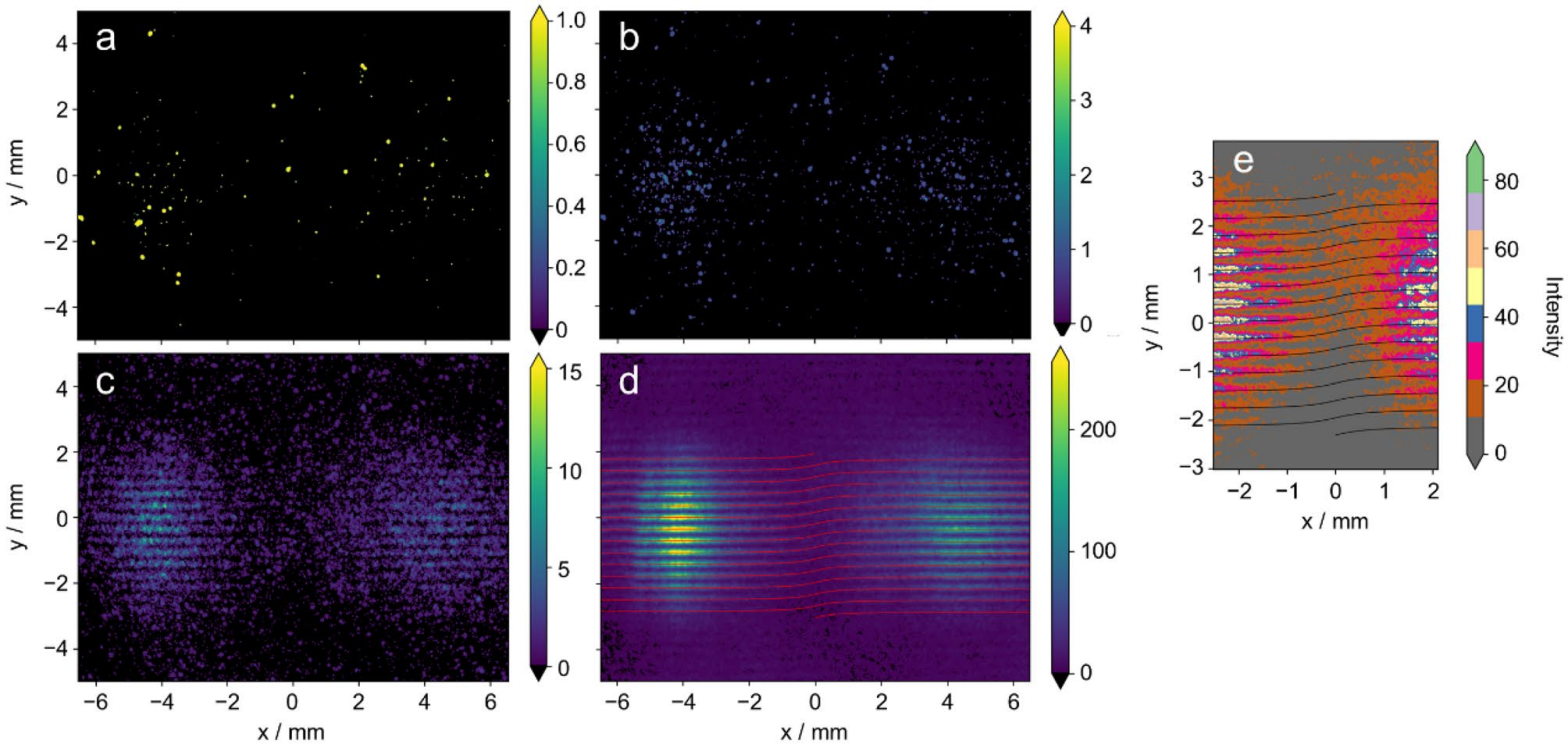
Images by photon-counting detection of Young’s double-slit interference with undulator vortex radiation. (**a**) Singe-shot image measured for ICCD camera with an exposure time of 200 µs. (**b**–**d**), Integrated images for (**b**) 5 shots, (**c**) 100 shots, and (**d**) 5000 shots. Interference fringes calculated using Eq. (1) are also drawn as red lines in (**d**) (*l* = 1). (**e**) Clear breaks/distortions of fringes are measured under counting regime by enlarging the central dark area in (**d**) with different color tones. These are in good agreement with the black curves obtained using the same equation.

Subsequently, we conducted image capturing using photon-counting detection with a gated intensified charge-coupled device (ICCD) camera, which can detect individual photons in the same way as the photon detection system used in Young's interference experiments in reference^22^. Figure 2a illustrates one of the 5000 shots obtained in total, where the gate width of the image intensifier (I.I.) was set to 200 μs. In the binarized representation of the single-shot image, approximately 130 photons are observed as bright spots, but there is no discernible regularity in their distribution, and the photons appear to be randomly scattered. Figure 2b exhibits an integrated image of five single shots, where the spots still appear to be randomly distributed. However, as the integration increases to 100 shots, interference fringes begin to emerge, and in Fig. 2c, clear interference fringes and dark breaks at the center become visible. After integrating 5000 shots (Fig. 2d), the interference fringes have fully developed, displaying a distinct pattern. The process of accumulating interference fringes by integrating seemingly random photon distributions can be observed in the Supplementary Movie. The cumulative movie provides a visual demonstration of the progressive formation of interference fringes through the accumulation of individual shots.

To ensure that the measurement was conducted in a single-photon regime, several considerations were taken into account. The UVSOR synchrotron generates radiation pulses with a duration of 100 ps at a repetition rate of 90 MHz. Consequently, a 0.1 ns wide light pulse arrives at the double slits every 11 ns, equivalent to a 30 mm light pulse every 3.3 m. With the distance between the double slits and the ICCD camera's photocathode being 1 m, only one light pulse exists in the observation system at any given time. In this experiment, the storage ring was operated in an extremely low-current mode to generate very weak light pulses, which were then transmitted through an ultranarrow bandpass filter to achieve the experimental conditions that greatly reduced the number of photons. On average, 130 photons were detected per image with a gate width of 200 μs using the gated ICCD camera (Hamamatsu C11370-10-1) with a quantum efficiency of approximately 20%. Considering this efficiency, on average, one photon was detected every 28 synchrotron radiation (SR) pulses. Assuming a Poisson distribution for the number of photons in a radiation pulse, the probability of finding one photon in a pulse is 3.4 × 10^−2^, while the probability of finding two or more photons is 6.2 × 10^−4^, which is significantly lower. Furthermore, an ultranarrow bandpass filter (Alluxa 7057) was employed in the experiment to isolate and measure only the second harmonic radiation at 355.00 nm, which exhibits OV characteristics. The filter has a full width at half maximum of 0.17 nm. Considering the coherence length of a single photon under the measurement conditions (0.74 mm), which is considerably shorter than the optical pulse length of 30 mm, it can be inferred that the interaction between photons, arising from multiple photons within a single radiation pulse, is negligible. Therefore, we can be confident that the measurements were conducted in the single-photon regime.

In Fig. 2e, an enlarged view of the central part of the Young's interference fringes of the OV measured in the photon-counting regime is presented. It is evident that clear breaks and misalignment of the fringes are observed. The calculated thin lines, based on the experimental conditions using Eq. (1) as described in Fig. 1, are in excellent agreement with the measured results and accurately reproduce the distortions of the interference fringes. This provides strong evidence that the buildup of the interference fringes shown in Fig. 2 (and the Supplementary Movie) is a consequence of the fact that even a single photon possesses the property of an OV. Here, we would like to mention the contrast with a recent study that similarly observed the buildup of photoelectron interference using the same undulator^24^. In this study, vortex photons generated by a single undulator were observed to be spatially self-interfering with the double slits. On the other hand, in reference^24^, a pair of light wave packets generated by two tandem undulators ionize He atoms, and a resulting pair of photoelectron wave packets interfere with each other in time, which is observed as interference in the energy-domain.

**Figure 3:**
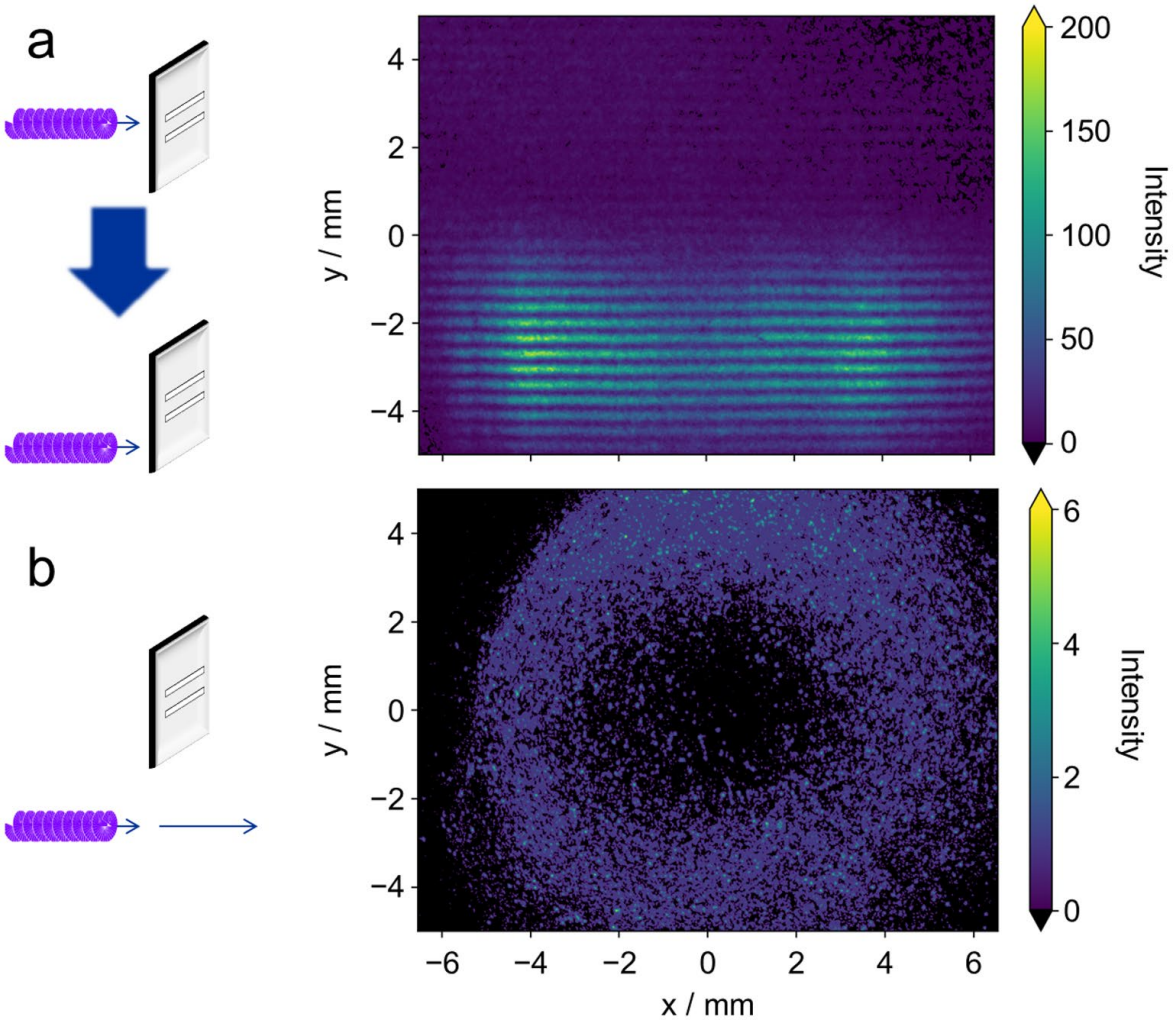
Images measured by different experimental conditions in the photon-counting regime. (**a**) 5000-shots-integrated interference image with the phase singularity of OV out of the double slits. (**b**) Single-shot image measured with I.I. gate width of 1 ms after removing the double slit.

To further verify the characteristics of the OV, the measurement was performed under the same experimental conditions but with a different height of the double slits, causing the optical axis (i.e., the phase singularity of OV) to be positioned outside the double slits. The resulting image obtained from 5000 shots is displayed in Fig. 3a. In this image, there are hardly any breaks or distortions in the center of the interference fringes, and the fringes exhibit a typical stripe pattern. This corresponds to interference fringes with a significantly smaller phase difference, as expected when the phase singularity of OV is not positioned between the double slits, thereby resembling the results of an interference experiment using ordinary plane waves. Furthermore, the nature of vortex radiation from the undulator in the photon-counting regime is further confirmed by completely removing the double slit, as shown in Fig. 3b. In this case, the resulting intensity distribution reveals a donut-shaped beam profile with the center missing. This distinctive feature is characteristic of vortex radiation, where the intensity at the center is zero due to the presence of a phase singularity. Comparing the intensity distribution in the transverse direction with Fig. 2d, it can be observed that the bright and dark areas coincide in almost the same region. From this, it can be deduced that the interference image of OV exhibits an eyeglass-shaped intensity distribution.

## Conclusion

The Young's double-slit interference experiments conducted using undulator vortex radiation in the photon-counting regime have successfully demonstrated that even a single photon possesses the characteristics of OV. The integration of seemingly randomly distributed single-shot images revealed the formation of interference fringes with the distinctive features of OV. This confirms that the harmonic radiation generated by the helical undulator retains the OV characteristics even at the level of single photons. Essentially, a single photon emitted spontaneously from a high-energy electron in spiral motion exhibits the inherent helical wavefront nature.

The characteristics of OV emitted from the undulator are not limited to the ultraviolet region but can also be observed in the extreme ultraviolet and X-ray regions. In these photon energy ranges, the spiral spacing becomes significantly shorter by several orders of magnitude compared to the ultraviolet region. This can lead to unique physical properties such as photoetching, where the interaction with materials becomes more pronounced. Moreover, the enhanced interaction with materials opens up possibilities for investigating and understanding new light–material interactions through novel optical transitions. Overall, undulator vortex radiation holds great potential for advancing X-ray diffraction and imaging, paving the way for new scientific discoveries and applications in these fields.

## Methods

### Experimental setup

The experiments were conducted at the BL1U undulator beamline of the UVSOR-III electron storage ring^23^ using an extremely low-current mode (typically 0.1 mA). As UVSOR-III is a low-energy SR source, BL1U allows for the direct extraction of undulator light in the visible and ultraviolet regions into atmosphere without the need for additional optical elements. Therefore, commercially available optical elements and CCD cameras could be used for the experiments. The APPLE-II undulator was operated in circularly polarized mode to generate harmonics that exhibit the characteristics of OVs. The electron beam executing a spiral motion in the undulator produced semimonochromatic fundamental light at 710 nm without the use of a monochromator. The undulator radiation was spatially coherent due to the narrow electron beam emittance in the operation mode, making it diffraction limited in the ultraviolet wavelength region^18^.

**Figure 4:**
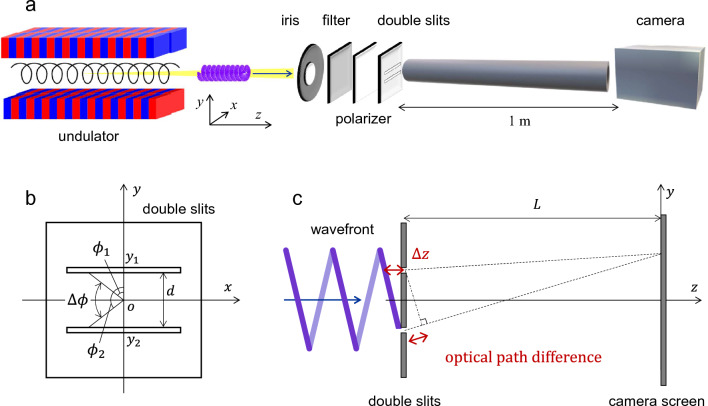
Experimental setup. (**a**) Schematic of Young’s double-slit experiment with helical-undulator-radiated second harmonic under photon-counting regime. (**b**) Coordinate for the double slit depicting the phase difference $\Delta \phi$ owing to the helical wavefront structure. (**c**) Optical path difference in Young’s double-slit experiment with OV.

Figure 4a depicts the schematic setup of the experiment. The circularly polarized radiation centered at 355 nm, corresponding to the second harmonic, was extracted from the vacuum beamline into atmosphere through a quartz window. The radiation passed through an iris, an ultranarrow interference bandpass filter (Alluxa 7057 354.72-0.15 OD5), and a polarizer before irradiating a double slit with a width of 0.1 mm and a separation of 1 mm. The iris and bandpass filter were employed to extract the central part of the radiation beam and remove the fundamental 710 nm light from the undulator radiation (actual measured specification of the filter is 355.00 nm center wavelength, a 0.17 nm full-width at half-maximum (FWHM), and wide-range OD5 out of the band). The polarizer, placed vertically, eliminated horizontally polarized radiation from the bending magnet and allowed only the undulator-derived light to pass through. By minimizing the use of optics, the experiment achieved the detection of very low photon counts, approaching single-photon counting regime while preserving the helical wavefront structure.

The photons that passed through the double slit were detected using two types of CCD cameras: a conventional one and a gated ICCD camera. The distance between the double slit and the photon-receiving plate of the camera was 1 m. Metal pipes were used to connect the double slit and the camera, and the setup was covered with black cloth to minimize stray light. During the experiment, the ceiling lights near the beamline were turned off. The gated ICCD camera system (Hamamatsu Photonics, C11370-10-1) used for photon-counting measurements consisted of an I.I. with a photosensitive area of 9.9 mm × 13.0 mm and a CCD camera with a resolution of 1024 pixels × 1344 pixels. The quantum efficiency of the ICCD camera at 355 nm was approximately 20%. The experimental data acquired with the ICCD camera involved capturing 5,000 images with an I.I. gate width of 200 µs. The data was saved as 16-bit TIFF files, and subsequent analysis, integration, and display were performed using Python. The obtained images were processed by binarization, converting elements below the threshold to zero and elements above the threshold to one, using a threshold value determined as the background level. The ImageJ program was utilized to measure the number of photon spots in the processed images.

### Double-slit interference of optical vortex

The electric field vector of light in Laguerre–Gaussian mode can be written as

2 $$\begin{array}{c}\overrightarrow{E}\left(x,y,z,t\right)\propto exp\left\{i\left(kz-\omega t+l\phi \right)\right\},\end{array}$$

where *k* is the wavenumber, $\omega$ is the angular frequency, $\phi$ is the azimuthal angle, and *l* is the angular quantum number (topological charge) in Cartesian coordinates with the propagation direction of light *z*. Thus, the isotropic surface (wavefront) at a given time is

3 $$\begin{array}{c}kz+l\phi =const.\end{array}$$

Equation (3) represents the helicity of wavefront around the *z*-axis.

In a standard Young's interference experiment involving plane waves, the phase of light remains constant as it passes through the double slits. Consequently, interference is observed based on the optical path difference between the distances from the double slits to the detection plate. However, in the case of OVs, the phase of light varies depending on the position of the double slits, leading to the generation of a new optical path difference. To establish a frame of reference, Cartesian coordinates are defined with the *x*-axis parallel to the slits, as depicted in Fig. 4a. The coordinates of the double slits, as observed from the *z*-axis direction, are illustrated in Fig. 4b. Assuming that the phase singularity of the light is located at the origin *O* between the two slits, the optical path difference $\Delta z$ for the OV passing through points (*x*, *y*_1_) and (*x*, *y*_2_) on the two slits at a given *x* can be expressed using Eq. (3) and the phase difference $\Delta \phi$ as follows:

4 $$\begin{array}{c}\Delta z=-\frac{l\Delta \phi }{k}=-\frac{l\lambda\Delta \phi }{2\pi },\end{array}$$

where $\lambda$ is the wavelength of the light. Let the azimuth angles at the two points on the slits be ${\phi }_{1}$ and ${\phi }_{2}$, respectively:

5 $$\begin{array}{c}\Delta \phi \equiv {\phi }_{1}-{\phi }_{2}={{\text{tan}}}^{-1}\left(\frac{x}{{y}_{1}}\right)-{{\text{tan}}}^{-1}\left(\frac{x}{{y}_{2}}\right).\end{array}$$

As shown in Fig. 4c, since the total optical path difference $\Delta s$ can be defined by the normal optical path difference after passing through the double slit and $\Delta z$,

6 $$\begin{array}{c}\Delta s=\frac{d}{L}y-\frac{l\lambda\Delta \phi }{2\pi },\end{array}$$

and bright fringes occur when $\Delta s=m\lambda$, where *m* is an integer, *x* and *y* are also the coordinates at the CCD camera screen, *d* ${(=y}_{1}-{y}_{2})$ is the distance between the slits, and *L* $(\gg d)$ is the distance from the slits to the screen. Thus, the interference fringes at the camera palate can be defined by the following equation:

7 $$\begin{array}{c}y=\frac{L\lambda }{d}\left(m+\frac{l}{2\pi }\Delta \phi \right)=\frac{L\lambda }{d}\left[m+\frac{l}{2\pi }\left\{{{\text{tan}}}^{-1}\left(\frac{x}{{y}_{1}}\right)-{{\text{tan}}}^{-1}\left(\frac{x}{{y}_{2}}\right)\right\}\right].\end{array}$$

### Supplementary Information

Supplementary Legends.

Supplementary Movie 1.

## Data Availability

All data that support the findings of this study are available from the corresponding author upon reasonable request.
