# Supplementary material for: Young’s double-slit experiment with undulator vortex radiation in the photon-counting regime
Source: Sci Rep. 2023 Dec 27;13:22962. doi: 10.1038/s41598-023-49825-4 (PMC10752875; doi:10.1038/s41598-023-49825-4)
Supplement: Supplementary file 1 — Supplementary Legends. [file 41598_2023_49825_MOESM1_ESM.docx]

**Supplementary Information**

**Young’s double-slit experiment with undulator vortex radiation in photon counting regime**

Shin-ichi Wada^1,2^, Hiroyuki Ohta^2^, Atsushi Mano^3^, Yoshifumi Takashima^3^, Masaki Fujimoto^4,3^, Masahiro Katoh^5,2,4^

^1^Graduate School of Advanced Science and Engineering, Hiroshima University, Higashi-Hiroshima 739-8526, Japan

^2^Faculty of Science, Hiroshima University, Higashi-Hiroshima 739-8526, Japan

^3^Synchrotron Radiation Research Center, Nagoya University, Nagoya, 464-8603, Japan

^4^Institute for Molecular Science, Okazaki 444-8585, Japan

^5^Hiroshima Synchrotron Radiation Center, Hiroshima University, Higashi-Hiroshima 739-0046, Japan

*Corresponding authors:

Shin-ichi Wada

E-mail address: wadasin@hiroshima-u.ac.jp

Masahiro Katoh

E-mail address: mkatoh@hiroshima-u.ac.jp

**Supplementary Material**

**Supplementary Movie S1. Visual demonstration of the progressive formation of Young's interference fringes with undulator vortex radiation measured in photon-counting regime.** Related to Fig. 2 (a)–(d).

The images were captured using a gated ICCD camera at the BL1U undulator beamline of the UVSOR-III using a quite low-current mode. The movie provides buildup of the interference fringes with dark breaks/distortions at the center by the accumulation of 5000 single-shot images where there is no discernible regularity in their distribution, and the photons appear to be randomly scattered.
